# Effects of oyster glycogen intramammary challenge on primiparous cow milk somatic cell counts, milk yields, and milk composition

**DOI:** 10.3168/jdsc.2023-0387

**Published:** 2023-10-03

**Authors:** B.D. Enger, C.S. Gammariello, M.X.S. Oliveira, P.H. Baker, K.M. Enger

**Affiliations:** Department of Animal Sciences, Ohio Agricultural Research and Development Center, The Ohio State University, Wooster, OH 44691

## Abstract

Mastitis is a common and costly disease in the dairy industry that reduces milk production in affected mammary glands. The local mechanisms that result in reduced milk production of affected mammary glands are incompletely understood; elucidation of these mechanisms is dependent on the use of hypothesis testing studies, but few experimental models exist. The objective of this study was to develop a mastitis challenge model, using a split udder design, to reduce milk yields by approximately 15% in udder halves challenged with oyster glycogen, a known inducer of leukocyte recruitment, relative to udder halves treated with saline. Four primiparous Holstein cows in mid lactation were used. One udder half of each cow was randomly selected and challenged with oyster glycogen (OYGLN), and the opposite udder half was treated with saline (SAL). Milk yields and components were measured at each milking (3×/d) for 3 d postchallenge. No signs of clinical mastitis were observed. Milk somatic cell scores, yields, and components were similar between OYGLN and SAL udder halves at time of challenge. Milk somatic cell scores markedly increased in OYGLN halves postchallenge and were greater than SAL halves for the duration of the trial. Lactose concentrations of OYGLN udder halves were transiently lower than in SAL udder halves, but protein concentrations were greater at 2 milkings postchallenge in OYGLN halves. Milk yields and energy-corrected milk yields did not differ between OYGLN and SAL udder halves overall, nor at any postchallenge milking. A single intramammary challenge of oyster glycogen was unsuccessful in eliciting a disparity in milk yields between challenged and saline control udder halves despite the marked leukocyte infiltration observed in the former. These results indicate an incomplete understanding of how milk yields are reduced in mammary glands affected by subclinical mastitis and that transient somatic cell recruitment and infiltration alone do not directly reduce milk yields during subclinical mastitis.

## Highlights

• This is the first study to assess milk yields in an udder half model using agents intended to elicit subclinical mastitis.
• Oyster glycogen infusion greatly increased milk somatic cell score but did not noticeably influence milk yield.
• Acute, temporal, immune cell infiltration may not directly produce the milk yield losses observed during subclinical mastitis.

## Graphical Abstract

**Figure:**
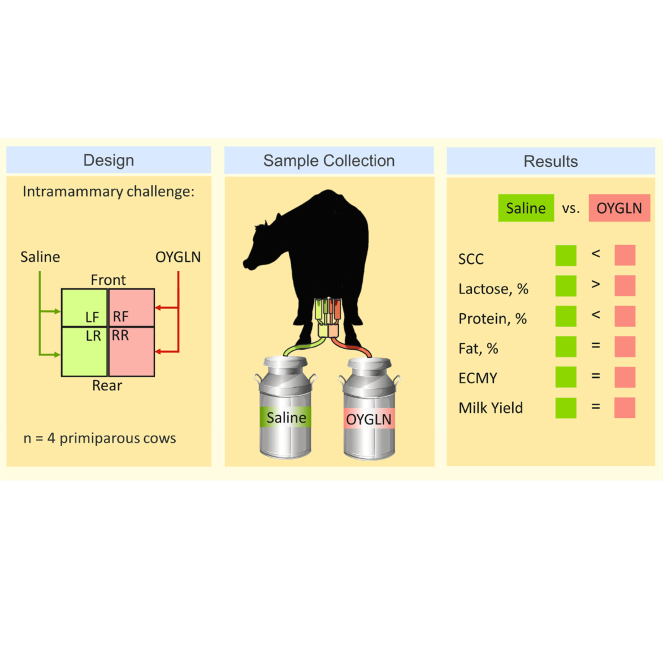
**Summary:** Four primiparous Holstein cows in mid lactation were infused with oyster glycogen (OYGLN) and saline in randomly selected udder halves. Udder half milk composition and milk yields were measured for 3 days postchallenge. Milk of udder halves treated with OYGLN had greater somatic cell counts (SCC) and protein percentages, but reduced concentrations of lactose. Milk fat percentages, milk yields, and energy-corrected milk yields (ECMY) were unaffected by udder half treatments. The results indicate an incomplete understanding of how milk yields are reduced during subclinical mastitis.

Mastitis is one of the most common and expensive diseases in the US and global dairy industries, and the reduced milk yield of affected cows accounts for a significant portion of monetary losses. Post hoc association studies have identified a negative relationship between test day SCC and milk yields (Jones et al., 1984; Hand et al., 2012), suggesting that the degree of immune cell recruitment and infiltration drives milk yield losses. Several physiological mechanisms have been proposed to explain why milk yield is reduced during mastitis in affected mammary glands, but the exact mechanisms remain unsettled. Elucidating the local mammary gland mechanisms that reduce milk synthesis and secretion during subclinical and mild clinical mastitis are contingent on hypothesis testing models that have yet to be established.

Mastitis challenge studies routinely use live bacteria or noninfectious agents to elicit an immune response. Classic examples of the latter include LPS, lipoteichoic acid, heat-inactivated or formalin-fixed bacteria, and oyster glycogen. Advantages of using noninfectious agents include (1) the limited risk of introducing an infectious agent into the herd, (2) inducing a temporal inflammatory state that should resolve without intervention, and (3) mechanistically isolating tissue and milk responses in the host from contributions of microbial metabolism and pathogenesis.

Investigations of local mammary gland factors often use a split plot/udder design because the design controls for between-animal variation. The design eliminates the effects of differing environmental, nutritional, and genetic factors between animals by isolating local responses to individual mammary glands or udder halves, allowing for treatment comparisons within animal (Wall and McFadden, 2012). Indeed, numerous mastitis challenge studies (Shuster et al., 1991; Shangraw et al., 2020) have used this study design, but to our surprise, only a single study (Fox et al., 1986) could be identified by us that evaluated milk yield responses to intramammary challenges using agents other than LPS, which classically produces a robust clinical response. Fox et al. (1986) reported that intramammary infusion of oyster glycogen, an inducer of leukocyte recruitment, may reduce a challenged quarter's milk yield, but Fox et al. only challenged 1 quarter/cow and compared it with the healthy contralateral control quarter; milk yield reductions were reported to exist, but differences were not overtly apparent. The objective of this study was to develop a mastitis challenge model where a disparity in udder half milk yield is elicited by treating one udder half with oyster glycogen to reduce milk yield approximately 15% relative to saline-treated udder halves.

The use of animals and procedures was reviewed and approved by The Ohio State University Institutional Animal Care and Use Committee (protocol 2022A00000020).

Clinically healthy, mid-lactation primiparous Holstein cows (n = 4) were selected from the Ohio State University Krauss Dairy based on previous SCC and milk yield. No sample size calculation was performed, but the number of animals utilized was decided after evaluating SCC of previous oyster glycogen challenge studies (Paape et al., 1977; Fox et al., 1986) and comparing the resulting SCC with expected milk yield losses that are associated with different thresholds (Jones et al., 1984; Hand et al., 2012). Mean daily milk yield, DIM, and SCC of cows at enrollment were 39.9 kg (SD = 6.7 kg), 174 d (SD = 17 d), and 27,000 cells/mL (SD = 25,000 cells/mL), respectively. Cows were acclimated to tiestalls for at least 1 wk before they were moved to new tiestalls; cows were fed twice daily, and milking frequency was increased from 2× to 3×/d for 3 d before the beginning of the 3-d study. Increased milking frequency was applied to more precisely identify when leukocyte recruitment and infiltration occurred and better identify when any changes in milk yield and composition may occur. Cows were milked in the tiestalls with a Surge RX (GEA North America, Columbia, MD) quarter milker to separate milk from the right and left udder halves into 2 separate buckets. Udder half milk yields were recorded at each milking and did not differ within each cow by more than 0.5 kg before study commencement.

Beginning at the start of the trial, immediately after first milking, 2 cows were randomly selected and the quarters of 1 udder half of each cow were both infused with 100 mg of oyster glycogen (**OG100**). The quarters of the opposite udder half were infused with saline (**SAL**). The remaining 2 cows were similarly treated, but a higher oyster glycogen dosage was utilized (125 mg/quarter (**OG125**) to determine if a greater dosage was needed to achieve the desired disparity in udder half milk yield. All treatment allocations were random. Udder half milk yields and milk samples were obtained immediately before challenge and at all subsequent milkings. Milk SCC and components were measured by a commercial DHIA laboratory via a Bentley Fourier Transform Spectrometer and Flow Cytometer (Bentley Instruments, Chaska, MN). At each milking, all quarters were fore-stripped and examined to identify signs of clinical mastitis; rectal temperatures were also obtained for 48 h postchallenge. Because individual quarters can take different times to finish milking, the milking unit was not removed until all quarters had concluded milking as visually determined by a nearly complete absence of milk flow.

All intramammary infusions followed aseptic procedures described previously (Enger et al., 2018) and utilized the partial insertion method (Boddie and Nickerson, 1986). Infused oyster glycogen (Sigma-Aldrich Inc., St. Louis, MO) was sterilized using absolute ethanol following the methods of Fox et al. (1986) before being dissolved in sterile PBS. Saline and oyster glycogen infusate volumes were 20 mL/quarter.

Milk SCC were transformed into SCS to satisfy assumptions of equal variance; SCC <12,500 cells/mL were increased to this minimum value because lesser counts cannot be transformed. Energy-corrected milk yields (**ECMY**) were computed as described previously (Western et al., 2020). Milk components, milk yields, and ECMY were analyzed in separate models using PROC MIXED (SAS 9.4, SAS Institute Inc., Cary, NC). Fixed effects of the model were udder half treatment (n = 3), time point (n = 9), and their interaction; cow nested within treatment was a random effect and measures were repeated on cows nested within treatment over time. Least squares means were computed and contrasted using Fisher's least significant difference test.

We did not observe any signs of clinical mastitis throughout the trial, and no cows developed a febrile response. Milk SCS were differentially affected by udder half treatments over time (*P* < 0.001; Figure 1A). Milk SCS were similar among udder half treatments at challenge (*P* = 0.3), but SCS of OG100 and OG125 udder halves increased and were markedly greater than SAL halves at all subsequent milkings (*P* ≤ 0.001). Milk SCS of OG100 halves did not significantly differ from OG125 halves any time postchallenge (*P* ≥ 0.17) but were consistently lower. Concentrations of lactose (*P* = 0.30), fat (*P* = 0.97), and protein (*P* = 0.94) were unaffected by udder half treatments and the interactive effect of treatment by time (*P* ≥ 0.33; Figure 1B–D). Similarly, milk yield (*P* = 0.64) and ECMY (*P* = 0.4) did not differ among udder half treatments and were not influenced by the effect of treatment interacting with time (*P* ≥ 0.87; Figure 1E–F).

**Figure 1:**
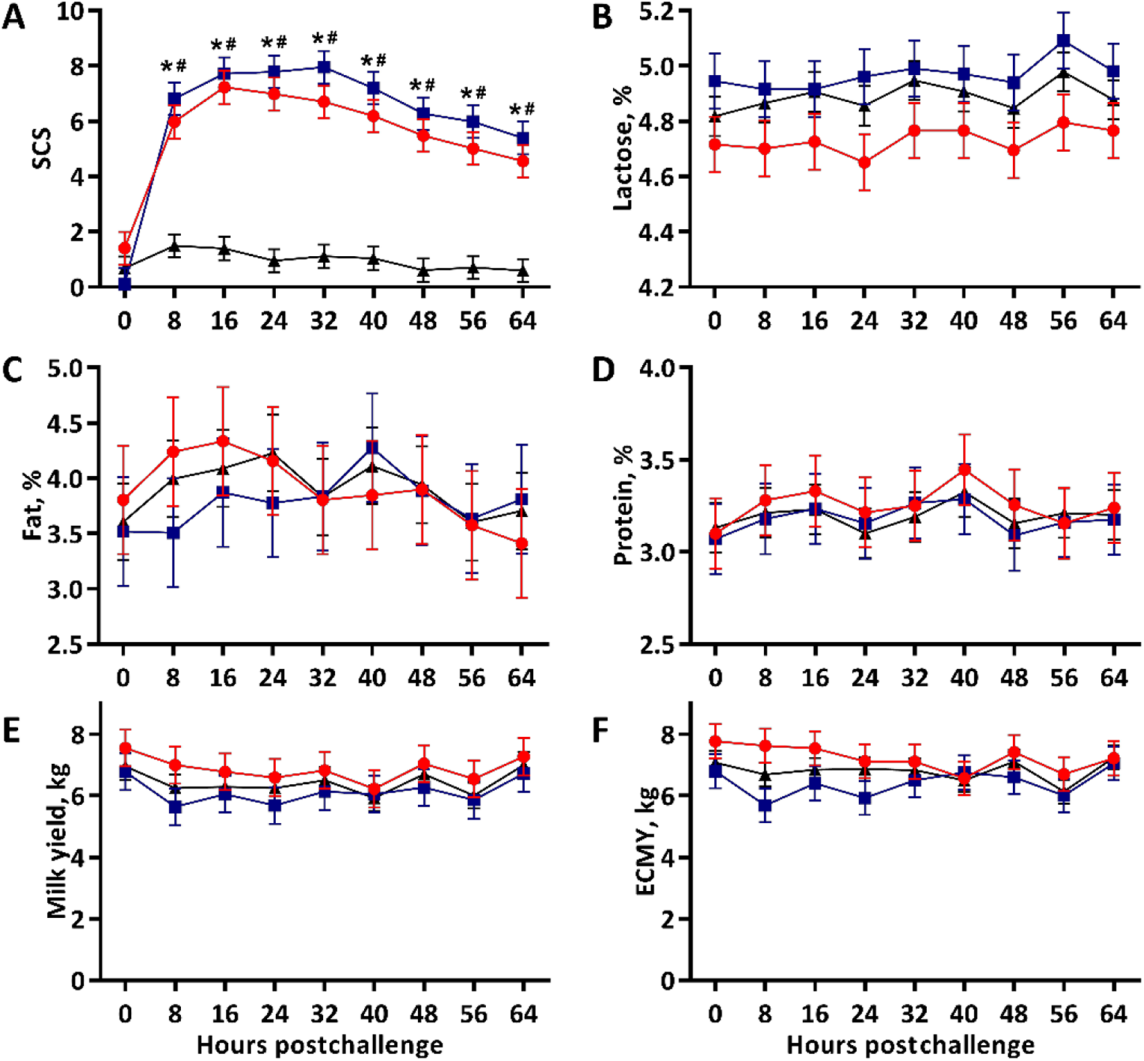
Mean milk SCS (A), lactose percentages (B), fat percentages (C), protein percentages (D), milk yields (E), and energy-corrected milk yields (ECMY, F) of udder halves that received intramammary infusions of saline (black triangles, n = 4), 100 mg of oyster glycogen/quarter (red circles, n = 2), or 125 mg of oyster glycogen/quarter (blue squares, n = 2). Error bars denote the SEM. * indicates *P* ≤ 0.05 for saline versus 100 mg oyster glycogen comparisons within time point, and # indicates *P* ≤ 0.05 for saline versus 125 mg oyster glycogen comparisons within time point.

This study was limited in power because it was strictly intended to identify an oyster glycogen dosage capable of temporally reducing udder half milk approximately 15%. To reduce the likelihood of a type 2 error, a confirmatory statistical analysis was conducted where all oyster glycogen–challenged halves, regardless of dose, were treated as 1 treatment, **OYGLN**. Fixed effects were udder half treatment (n = 2), time point (n = 9), and their interaction; cow was a random effect and measures were repeated for each treatment by cow combination over time. The results largely align with the first analysis. Milk SCS of OYGLN and SAL halves were similar at challenge (*P* = 0.98) but were greater in OYGLN halves at all postchallenge milkings (*P* ≤ 0.003; Figure 2A). The effects of udder half treatment and treatment interacting with time did not affect milk fat percentages (*P* ≥ 0.56; Figure 2C), milk yield (*P* ≥ 0.58; Figure 2E), or ECMY (*P* ≥ 0.94; Figure 2F). Conversely, lactose percentages were transiently reduced in OYGLN halves (Figure 2B), being lower than SAL halves from 8 to 32 h postchallenge (*P* ≤ 0.09). Milk protein percentages followed a less pronounced response to challenge as OYGLN protein percentages were only greater than SAL halves at 24 and 32 h postchallenge (*P* = 0.04 and 0.11, respectively; Figure 2D).

**Figure 2:**
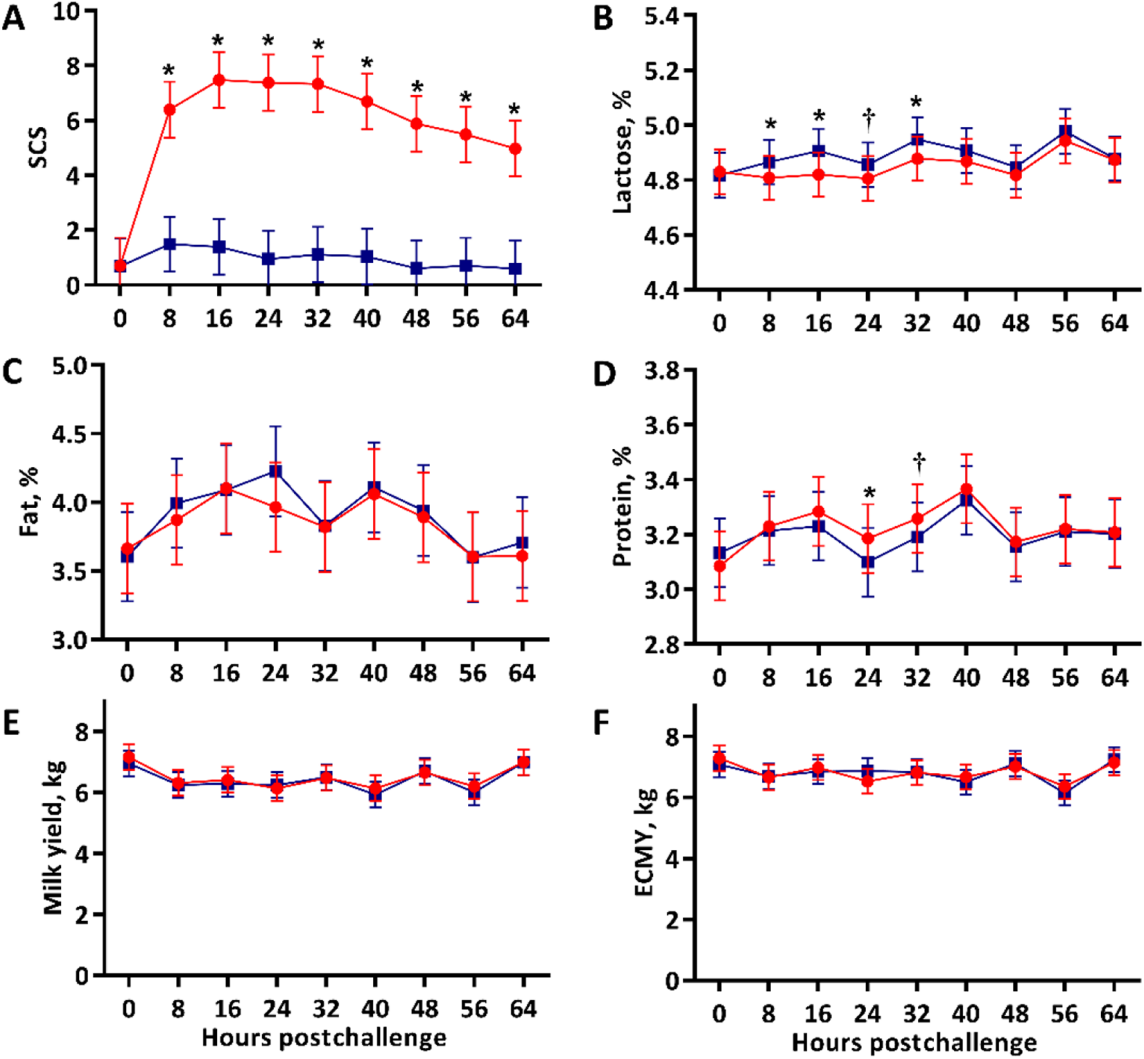
Mean milk SCS (A), lactose percentages (B), fat percentages (C), protein percentages (D) milk yields (E), and energy-corrected milk yields (ECMY; F) of udder halves that received intramammary infusions of saline (blue squares, n = 4) or oyster glycogen (red circles, n = 4). Error bars denote the SEM. † indicates that means differ at *P* ≤ 0.11 within time point, and * indicates that means differ at *P* ≤ 0.05 within time point.

The changes in milk composition detected in the confirmatory analysis were expected and indicate limited statistical power of the first analysis. Changes in milk composition resulting from mastitis are well established (Akers and Thompson, 1987; Pyörälä, 2003) and align with the observations here. Most strikingly, however, is the absence of a milk yield or ECMY response to oyster glycogen challenge in both statistical analyses. This result was unexpected and noteworthy, especially when it is recognized the mean peak SCC response of OY100 and OY125 halves exceeded 1,900,000 and 3,000,000 cells/mL, respectively. Reasons for the lack of a milk yield response are unclear, but several factors may have contributed to this result.

First, the immunogenic properties of oyster glycogen are incompletely understood. It has been hypothesized that proteins in oyster glycogen stimulate leukocyte recruitment (Anderson et al., 1985), but we propose that the glycogen may be the chief inducer of leukocyte recruitment. Glycogen may act as a damage-associated molecular pattern because glycogen is seldom extracellular, and the host may recognize its extracellular presence to be indicative of cell lysis and tissue damage. Indeed, secretory epithelial cells contain glycogen, and glycogen is highly abundant in the epithelial cells lining the teat and gland cisterns (Reid and Chandler, 1973), 2 regions that would be first exposed, and highly affected by mastitis pathogens. The absence of pathogens or infection-induced tissue damage (or both) may have negated leukocyte activation and the initiation of inflammatory cascades. If this speculation is correct, immune cell recruitment may have been partially uncoupled from other inflammatory processes that occur during naturally occurring mastitis in this experimental setting.

Second, it may be that milk yield losses resultant of subclinical mastitis materialize after longer timeframes, and our 3-d challenge trial was of insufficient duration to observe a milk yield response. Nickerson (1980) reported that challenging 1 quarter/cow with *Staphylococcus aureus* reduced cow milk yields an average of 0.35 kg/d, but a clear pattern of milk yield reductions was not evident until 8 d postchallenge. Inferences from studies evaluating intramammary devices further support the notion that extended periods (i.e., wk to mo) of leukocyte recruitment and infiltration are necessary to elicit milk yield responses; quarters administered intramammary devices have greater SCC and exhibit reduced milk yields ranging from 0.1 to 0.3 kg/d (Paape and Weinland, 1988), to an average of 0.6 kg/d that was estimated over an entire lactation (Jaster et al., 1982). Ultimately, further research is needed to determine if duration of leukocyte recruitment and infiltration significantly affects milk yields or how long infiltration is needed to elicit milk yield reductions.

Third, a greater SCC response may have been needed to elicit the desired disparity in milk yield. Curiously though, others (Wellnitz et al., 2013; Wall et al., 2016) have reported that SCC alone do not directly align with the degree of changes in milk composition and tight junction integrity (milk yields were not measured), indicating that factors other than the level of leukocyte recruitment influence inflammation severity. Accordingly, it is uncertain if simply increasing the degree of leukocyte recruitment would have altered the outcomes observed here in response to oyster glycogen challenge.

Last, milk yield losses related to SCC are significantly influenced by parity, with multiparous cows yielding greater milk yield losses than primiparous cows (Hand et al., 2012). We purposefully chose to use primiparous cows for this first study to remove any potential effects of previous lactations and dry periods that may have influenced pre- and postchallenge udder half milk yields. Accordingly, our use of primiparous cows may have reduced our ability to detect milk yield responses, but the results presented in Figures 2E and 2F do not support the notion that a change in milk yield may have existed and insufficient power prevented differences from materializing. Still, it remains unknown if multiparous cows would have responded differently to oyster glycogen challenge.

This study has important limitations that need be considered. While we, and others (Yoder et al., 2020), chose to increase milking frequency to better capture temporal changes in milk yield and composition, it remains unknown if increasing milking frequency altered any potential milk component and yield responses observed here. Additionally, the relocation of cows to milk-in-place tiestalls may have affected feeding behavior and subsequently affected milk production. Even though we applied an adaption period and chose to use an udder half model to reduce the effects management changes could have on our measures of interest, others may consider minimizing changes to animal management before studies that scrutinize potential changes in milk composition and yield to reduce potential confounding effects.

In conclusion, this was the first study to assess potential milk yield losses in an udder half model with agents intended to elicit temporal subclinical mastitis. The data of this pilot study illustrate an incomplete understanding of how milk yield is reduced during subclinical mastitis. The rapid influx of somatic cells resulting from a single oyster glycogen challenge elicited minor changes in lactose and protein concentrations but failed to noticeably reduce milk yield and ECMY of OYGLN-treated udder halves relative to SAL halves. These results indicate the isolated physiologic response of marked, transient somatic cell recruitment and infiltration does not significantly reduce milk yield of the affected mammary gland. Accordingly, factors other than transient, acute somatic cell recruitment and infiltration are posited to more greatly contribute to the observed milk yield reductions that result during naturally occurring subclinical mastitis.

## References

[bib1] Akers R.M., Thompson W. (1987). Effect of induced leucocyte migration on mammary cell morphology and milk component biosynthesis. J. Dairy Sci..

[bib2] Anderson K.L., Hemeida N.A., Frank A., Whitmore H.L., Gustafsson B.K. (1985). Collection and phagocytic evaluation of uterine neutrophilic leukocytes. Theriogenology.

[bib3] Boddie R.L., Nickerson S.C. (1986). Dry cow therapy: Effects of method of drug administration on occurrence of intramammary infection. J. Dairy Sci..

[bib4] Enger B.D., Crutchfield C.E., Yohe T.T., Enger K.M., Nickerson S.C., Parsons C.L.M., Akers R.M. (2018). *Staphylococcus aureus* intramammary challenge in non-lactating mammary glands stimulated to rapidly grow and develop with estradiol and progesterone. Vet. Res..

[bib5] Fox L.K., Timms L.L., Schultz L.H. (1986). Changes in bovine milk secretion following intramammary infusions of concanavalin A, oyster glycogen, or water. J. Dairy Sci..

[bib6] Hand K.J., Godkin A., Kelton D.F. (2012). Milk production and somatic cell counts: A cow-level analysis. J. Dairy Sci..

[bib7] Jaster E.H., Smith A.R., McPherron T.A., Pedersen D.K. (1982). Effect of an intramammary polyethylene device in primiparous dairy cows. Am. J. Vet. Res..

[bib8] Jones G.M., Pearson R.E., Clabaugh G.A., Heald C.W. (1984). Relationships between somatic cell counts and milk production. J. Dairy Sci..

[bib9] Nickerson S.C. (1980).

[bib10] Paape M.J., Pearson R.E., Wergin W.P., Guidry A.J. (1977). Enhancement of chemotactic response of polymorphonuclear leukocytes into the mammary gland and isolation from milk. J. Dairy Sci..

[bib11] Paape M.J., Weinland B.T. (1988). Effect of abraded intramammary device on milk yield, tissue damage, and cellular composition. J. Dairy Sci..

[bib12] Pyörälä S. (2003). Indicators of inflammation in the diagnosis of mastitis. Vet. Res..

[bib13] Reid I.M., Chandler R.L. (1973). Ultrastructural studies on the bovine mammary gland with particular reference to glycogen distribution. Res. Vet. Sci..

[bib14] Shangraw E.M., Rodrigues R.O., Witzke M.C., Choudhary R.K., Zhao F.Q., McFadden T.B. (2020). Intramammary lipopolysaccharide infusion induces local and systemic effects on milk components in lactating bovine mammary glands. J. Dairy Sci..

[bib15] Shuster D.E., Harmon R.J., Jackson J.A., Hemken R.W. (1991). Suppression of milk production during endotoxin-induced mastitis. J. Dairy Sci..

[bib16] Wall E.H., McFadden T.B. (2012). Triennial Lactation Symposium: A local affair: How the mammary gland adapts to changes in milking frequency. J. Anim. Sci..

[bib17] Wall S.K., Hernandez-Castellano L.E., Ahmadpour A., Bruckmaier R.M., Wellnitz O. (2016). Differential glucocorticoid-induced closure of the blood-milk barrier during lipopolysaccharide- and lipoteichoic acid-induced mastitis in dairy cows. J. Dairy Sci..

[bib18] Wellnitz O., Arnold E.T., Lehmann M., Bruckmaier R.M. (2013). Short communication: Differential immunoglobulin transfer during mastitis challenge by pathogen-specific components. J. Dairy Sci..

[bib19] Western M.M., de Souza J., Lock A.L. (2020). Milk production responses to altering the dietary ratio of palmitic and oleic acids varies with production level in dairy cows. J. Dairy Sci..

[bib20] Yoder P.S., Huang X., Teixeira I.A., Cant J.P., Hanigan M.D. (2020). Effects of jugular infused methionine, lysine, and histidine as a group or leucine and isoleucine as a group on production and metabolism in lactating dairy cows. J. Dairy Sci..

